# Arachidonic Acid Drives Postnatal Neurogenesis and Elicits a Beneficial Effect on Prepulse Inhibition, a Biological Trait of Psychiatric Illnesses

**DOI:** 10.1371/journal.pone.0005085

**Published:** 2009-04-08

**Authors:** Motoko Maekawa, Noriko Takashima, Miho Matsumata, Shiro Ikegami, Masanori Kontani, Yoshinobu Hara, Hiroshi Kawashima, Yuji Owada, Yoshinobu Kiso, Takeo Yoshikawa, Kaoru Inokuchi, Noriko Osumi

**Affiliations:** 1 Department of Developmental Neurobiology, Tohoku University Graduate School of Medicine, Sendai, Japan; 2 Laboratory for Molecular Psychiatry, RIKEN Brain Science Institute, Saitama, Japan; 3 Mitsubishi Kagaku Institute of Life Sciences (MITILS), Tokyo, Japan; 4 CREST, Japan Science and Technology Corporation (JST), Kawaguchi, Japan; 5 Saitama Institute of Technology, Saitama, Japan; 6 Institute for Health Care Science, Suntory Co. Ltd., Osaka, Japan; 7 Department of Organ Anatomy, Yamaguchi University Graduate School of Medicine, Yamaguchi, Japan; 8 Graduate School of Environment and Information Sciences, Yokohama National University, Yokohama, Japan; Chiba University Center for Forensic Mental Health, Japan

## Abstract

Prepulse inhibition (PPI) is a compelling endophenotype (biological markers) for mental disorders including schizophrenia. In a previous study, we identified *Fabp7*, a fatty acid binding protein 7 as one of the genes controlling PPI in mice and showed that this gene was associated with schizophrenia. We also demonstrated that disrupting *Fabp7* dampened hippocampal neurogenesis. In this study, we examined a link between neurogenesis and PPI using different animal models and exploring the possibility of postnatal manipulation of neurogenesis affecting PPI, since gene-deficient mice show biological disturbances from prenatal stages. In parallel, we tested the potential for dietary polyunsaturated fatty acids (PUFAs), arachidonic acid (ARA) and/or docosahexaenoic acid (DHA), to promote neurogenesis and improve PPI. PUFAs are ligands for Fabp members and are abundantly expressed in neural stem/progenitor cells in the hippocampus. Our results are: (1) an independent model animal, *Pax6* (+/−) rats, exhibited PPI deficits along with impaired postnatal neurogenesis; (2) methylazoxymethanol acetate (an anti-proliferative drug) elicited decreased neurogenesis even in postnatal period, and PPI defects in young adult rats (10 weeks) when the drug was given at the juvenile stage (4–5 weeks); (3) administering ARA for 4 weeks after birth promoted neurogenesis in wild type rats; (4) raising *Pax6* (+/−) pups on an ARA-containing diet enhanced neurogenesis and partially improved PPI in adult animals. These results suggest the potential benefit of ARA in ameliorating PPI deficits relevant to psychiatric disorders and suggest that the effect may be correlated with augmented postnatal neurogenesis.

## Introduction

The etiologies of psychiatric illnesses are largely unknown. Recently, biological traits relevant to psychiatric illnesses called “endophenotypes” have gained attention. Among them, deficits in prepulse inhibition (PPI) have been well documented across mental disorders including schizophrenia [1], [2]. PPI is the normal suppression of a startle response when a low intensity stimulus eliciting little or no behavioral response, immediately precedes an unexpected stronger startling stimulus [1]. Biologically, PPI is deemed to be a reflection of sensory motor gating functions within the central nervous system. The experimental advantage of PPI is that it is evaluable in animals. It is known that specific brain regions, including the hippocampus, cortex, amygdala and nucleus accumbens, form a circuit for PPI [3] (Figure S1), with hippocampal involvement being well studied. For instance, Weinberger and his colleagues reported that when rats received ventral hippocampal ablation during the early postnatal period, they exhibited PPI defects at the later adult stage [4].

In our recent study, we identified *Fabp7*, *that* encodes a brain-type fatty acid binding protein (also known as brain lipid binding protein; BLBP) as a gene responsible for PPI in mice and showed that human *FABP7* is a susceptibility gene for schizophrenia [5]. *Fabp7*-deficient mice did not show gross anatomical brain abnormalities, but interestingly, neurogenesis is impaired in the hippocampus. A similar biological cascade of “decreased neurogenesis” “impaired PPI” and “genetic association with schizophrenia” has been reported for *Npas3* [6]–[8] and *Neuregulin1* [9]–[11].

Here, we further address the question of whether decreased neurogenesis not only in the fetal (this is probably true in gene ablated animals) but also the postnatal period may serve as a determinant for PPI deficits. Since examining gene knockout mice would not be able to distinguish these ‘critical period’ issues, we utilized both genetic and pharmacological approaches in the analyses of *Pax6*(+/−) rats (for the former approach) and normal rates treated with the anti-proliferative drug, methylazoxymethanol acetate (MAM) after birth (for the latter one). In parallel, we also examined whether the dietary administration of polyunsaturated fatty acids (PUFAs) such as docosahexaenoic acid (DHA) and arachidonic acid (ARA) enhance neurogenesis in the rat hippocampus and elicit improved PPI. The ultimate hope is for developing preventive measures for mental disorders. In this study, we analyzed rats as experimental animals, because our study contained nutritional (varying lipid-containing feed) and to some degree toxicological issues where the use of rats is legitimate (http://www.ich.org/cache/compo/276-254-1.html). We found that disturbed postnatal neurogenesis led to impaired PPI. In contrast, ARA specifically promoted hippocampal neurogenesis and mitigated PPI defects seen in *Pax6*(+/−) rats that display both debilitated neurogenesis and PPI.

## Materials and Methods

### Animals

Wild-type (from CLEA JAPAN, Tokyo, Japan) and *Pax6* (+/−) (both on a Sprague-Dawley background) rats were maintained at Tohoku University, Sendai, Japan and Mitsubishi Kagaku Institute of Life Sciences, MITILS, Tokyo, Japan. All animal experiments were carried out in accordance with the National Institute of Health guidelines for the care and use of laboratory animals, and were approved by The Committee for Animal Experiments in the aforementioned organizations.

### PPI measurements

Rats were tested in a startle chamber (SR-lab System, San Diego Instruments, San Diego, CA) positioned within a soundproof cabinet in a sound-attenuating room according to standard methodology [12]. A constant background white noise of 65 dB was presented throughout the test. To measure prepulse inhibition (PPI), rats were presented with a 68, 71 or 77 dB prepulse (pp68, pp71 or pp77 for 20 ms) followed by a 105 or 120 dB pulse (for 40 ms in length) 100 ms later. The percentage PPI of the startle response was calculated using the following formula: 100-[(SRPP/SR)×100], where SR denotes the startle response to the pulse stimulus, and SRPP denotes the startle response to the pulse with prepulse stimulus.

### MAM treatment and subsequent analyses

Four-week old wild-type rats were intraperitoneally injected with MAM (0, 5, 7, 10 or 15 mg/kg) once a day for 1 week. For 5-bromo-2-deoxyuridine (BrdU) analysis (see below), MAM treated 5 and 10-week old rats were given a single BrdU injection and sacrificed 2 hours later. For behavioral analyses, the MAM treated rats were handled for 1 week, and then subjected to PPI tests.

### PUFA diets

We prepared 3 different diets by altering the fatty acid composition of AIN-76A: control, ARA(+), DHA(+) and ARA(+)DHA(+) diets (Table 1). Dietary foods with ARA or DHA were stored at 4°C and shaded from the light to prevent oxidation and denaturation. In addition, they were not treated with gamma rays or autoclave sterilization. Wild-type and *Pax6*(+/−) rats were fed from postnatal day 2 (P2) with DHA or ARA-triglyceride supplementation until they were used for various analyses.

**Table 1:** Fatty acid composition of the modified AIN-76A diets used in this experiment.

Fatty acids		PUFA Diets			
Abbreviated notation	Conventional name	Control (%)	ARA(+) (%)	DHA(+) (%)	ARA(+)DHA(+) (%)
16:0	Palmitic acid	11.5	11.3	11.6	11.6
18:0	Stearic acid	2.2	2.9	2.4	2.9
18:1 n-9	Oleic acid	28.8	26.8	28.6	26.6
18:2 n-6	Linoleic acid	54.7	50.3	50.4	46.1
18:3 n-3	alpha-Linolenic acid	0.9	0.9	0.9	0.8
20:3 n-6	Dihomo-gamma-linolenic acid	0.0	0.3	0.0	0.3
20:4 n-6	Arachidonic acid (ARA)	0.0	4.0	0.0	4.0
22:6 n-3	Docosahexaenoic acid (DHA)	0.0	0.0	4.0	4.0
	Others	1.9	3.5	2.1	3.7
	Total (%)	100.0	100.0	100.0	100.0

### Immunohistochemistry

For the analyses of expression markers related to cell proliferation, at least five rats at P30 were examined. Rats were deeply anesthetized with sodium pentobarbital and then transcardially perfused with 4% paraformaldehyde and 0.5% picric acid in 0.01 M phosphate-buffered saline (PBS). The brains were removed and further immersion-fixed in the same fixative at 4°C for 2 hours. Coronal sections of 14 µm thickness were prepared using a cryostat (CM3050, Leica, Germany). The sections were washed with Tris-buffered saline containing Tween 20 (TBST; pH 7.4). For immunostaining, the cryostat sections were incubated at 4°C for 18 hours with primary antibodies. For detection of the antigen localization, sections were incubated at 4°C for 2 hours with appropriate secondary antibodies. Information on the primary and secondary antibodies and other reagents is listed in Table S1. Fluorescent signals were detected using a confocal laser-scanning microscope (LSM5 Pascal, Zeiss, Germany) or a fluorescent microscope (Axioplan-2, Zeiss, Germany).

### BrdU labeling analysis

P30 rats fed on either control or ARA(+) or DHA(+) or ARA(+)DHA(+) diets received intraperitoneal injections of BrdU (Sigma, St. Louis, MO) at 50 mg/kg body weight (10 mg/ml stock, dissolved in 0.9% saline), 3 times a day and were sacrificed after 1 day. Fourteen µm frozen sections were boiled in 0.01 M citric acid and incubated in 2 N HCl for 10 min at 37°C, and washed in 0.01 M PBS. For quantification analysis, sampling of BrdU-positive cells was done throughout the dentate gyrus (DG) in its rostrocaudal extension. Three consecutive sections (14 µm each) out of a total of 18 sections were used for counting, and the total number of positives was obtained by multiplying the value by 6 [13].

## Results

### PPI deficits in *Pax6*(+/−) rats

**Figure 1:**
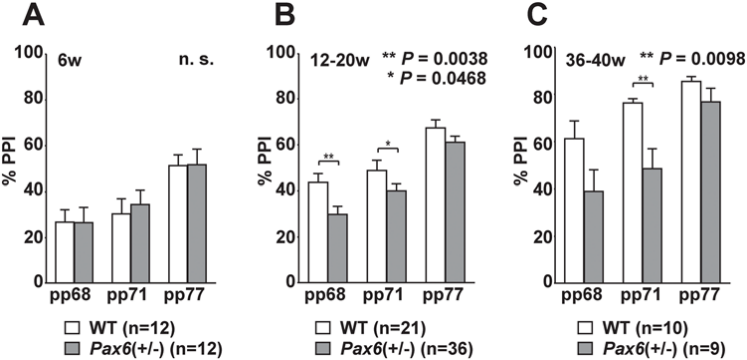
Impaired PPI in *Pax6*(+/−) rats. Wild type and *Pax6*(+/−) rats were tested for acoustic prepulse inhibition (PPI) of an acoustic startle response. Each rat was subjected to trials of different prepulse stimuli (pp) (68, 71, or 77 dB) with startle stimuli of 105 dB. (A) At 6 weeks, scores of PPI were not different between the wild type and *Pax6*(+/−) rats. n.s., not significant. (B) At 12–20 weeks, *Pax6*(+/−) rats showed a decrease in PPI at pp68 and pp71 compared with wild type rats. (C) Similar defects in PPI of *Pax6*(+/−) were also observed in rats aged over 36 weeks at pp71. Student *t*-test was used and error bars show mean±SE.

We examined *Small eye* rats (*rSey^2^*) that have a mutation in the transcription factor gene *Pax6* which controls neurodevelopment [14]. This animal model displays decreased hippocampal neurogenesis without any gross structural abnormalities in the brain [13]. To determine whether heterozygous *Pax6* mutant [*Pax6*(+/−)] rats have PPI defects (the homozygous mutantion is lethal), we scored PPI with acoustic stimuli (105 dB startle sound) at different postnatal stages (Figure 1A–1C). PPI levels were significantly lowered with 68 and 71 dB prepulse stimuli (*P* = 0.0038 and *P* = 0.0468, respectively; Figure 1B) at 12–20 weeks, and with 71 dB prepulse sound (*P* = 0.0098; Figure 1C) at 36–40 weeks in *Pax6*(+/−) rats, compared to wild-type rats. Decreased PPI was also observed when a pulse (startle sound) was set at 120 dB (data not shown). Interestingly, defects in PPI were not observed in juvenile rats (6 weeks; Figure 1A). This may correspond to the fact that schizophrenia, a typical mental illness that shows decreased PPI, develops after adolescence. The magnitude of the startle response itself was not significantly different between the wild type and *Pax6*(+/−) rats (Figure S2). Other behaviors were normal in *Pax6*(+/−) rats, except for rearing activity and the light-dark choice test (data not shown).

### Postnatal MAM treatment provokes reduction in neurogenesis and deficits in PPI

**Figure 2:**
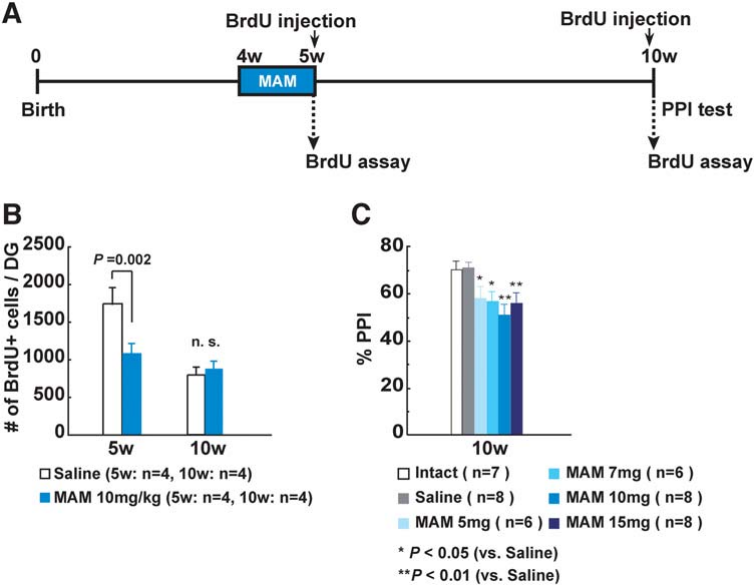
Decreased neurogenesis and impaired PPI in MAM-treated wild-type rats. (A), Experimental design for MAM treatment, BrdU assay and behavioral analyses in wild-type rats. (B), The number of BrdU-positive cells was decreased to 63% in the DG of MAM (10 mg/kg)-treated wild-type rats at 5 week, but did not differ between the two groups at 10 weeks of age. n.s., not significant. (C), PPI defects were observed at pp68 with a startle stimuli of 105 dB at 10 weeks in MAM-treated rats. Student *t*-test (B) or Fisher's PLSD test (C) was performed and error bars show mean±SE.

To explore the causative relationship between postnatal neurogenesis and PPI outcome in-depth, we used the drug MAM, a known attenuator of neurogenesis [15]. We administered the MAM to juvenile wild-type rats for 1 week (from at 4 to 5 weeks), and examined neurogenesis (at 5 and 10 weeks) and scored PPI (at 10 weeks) (Figure 2A and Figure S3A). The MAM-treated rats showed a transient decrease (63% of the control value) in hippocampal neurogenesis at 5 weeks (Figure 2B), then recovered neurogenesis to control levels by 10 weeks (Figure 2B). But interestingly, they displayed impaired PPI (at pulse levels of both 105 dB and 120 dB) at 10 weeks (Figure 2C and Figure S3A, S3B, S3C). We could not detect any differences in general activity measured by the open-field test at 10 weeks (data not shown). These results show that disturbances of postnatal neurogenesis, at least during the juvenile period, could evoke PPI deficits later in adulthood, without affecting locomotor activity. In addition, this is the first study demonstrating that PPI deficits can be induced by MAM treatments not only at fetal [16] but also at postnatal stages.

### Administration of arachidonic acid increases postnatal neurogenesis in the rat hippocampus

We thought that if we could foster hippocampal neurogenesis using dietary supplements, we could ameliorate PPI deficits, a biological marker relevant to mental disorders. We tested PUFAs because: (1) Fabp7 and another Fabp member Fabp5, can bind PUFAs [17], [18], and are abundantly expressed in neural stem/progenitor cells from fetal [19], [20] to adult stages (Figure S4A, S4B, S4C, S4D, S4E, S4F); and (2) Fabp7 is required for proliferation of neuroepithelial cells [19]. For the PUFAs, we chose the typical *n-3* and *n-6* lipids, DHA and ARA, respectively, and prepared dietary pellets containing these PUFAs (Table 1). PUFAs can be absorbed into the digestive organs and blood stream, and subsequently secreted into breast milk [21], [22]. When rat pups suck breast milk from their mothers treated with PUFAs, it is expected that PUFAs will be transmitted to body organs including the brain via the blood-brain barrier of the pups. Data presented below confirms that this occurred (see section below).

**Figure 3:**
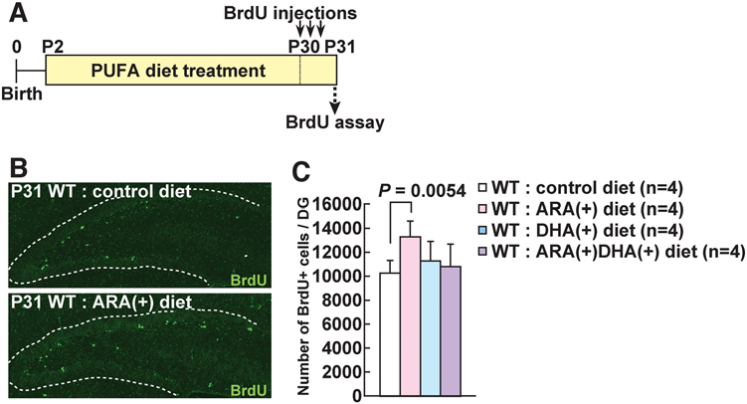
Effects on cell proliferation in the DG of ARA(+) and/or DHA(+) diet-treated wild-type rats. (A), Experimental design of the BrdU assay in PUFA (polyunsaturated fatty acid)-treated wild-type rats. Rat pups and their mothers were fed with varying PUFA-containing food from P2 to P31. They were sacrificed at 1 day after intraperitoneal BrdU injections. (B), The number of BrdU-positive cells was increased in the DG of ARA(+) diet-treated wild-type rats (lower panel) compared to those of control diet-treated rats (upper panel) at 1 day after BrdU injections. (C), The number of BrdU-positive cells in the DG of ARA(+) diet-treated wild-type rats was 32% higher than control diet-treated rats, while DHA(+) and ARA(+)DHA(+) diet-treated wild-type rats showed non-significant increase (+9% and +5%, respectively).

Rat pups from P2 and older and their mothers were fed with control, ARA(+), DHA(+), ARA(+)DHA(+) diets for 4 weeks (Figure 3A). We first measured the ratio of DHA and ARA in the hippocampus of the treated rat pups at P31, and confirmed that the ratio of ARA/DHA increased or decreased according to the administration of ARA or DHA, respectively (Text S1, Figure S5A, S5B). Next, the PUFA-treated rats were intraperitoneally injected with BrdU three times at day 30 (P30) and sacrificed one day after BrdU injections (at P31) (Figure 3A). We compared the total number of BrdU+ cells in the dentate gyrus (DG) of the control diet-, ARA(+) diet-, DHA(+) diet-, ARA(+)DHA(+) diet-treated rats. A 32 percent increase in the total number of BrdU-labeled cells was observed in ARA(+) diet-treated rats compared to control diet-treated rats (Figure 3B and 3C), whereas there were no significant differences between the control diet and DHA(+) diet- and between control diet and ARA(+)DHA(+) diet-treated rats (Figure 3C), although a trend of slight increase was seen in the DHA(+) diet- and to lesser extent ARA(+)DHA(+) diet-treated rats. We further investigated whether neural stem/progenitor cells in the DG were increased in ARA(+) diet-treated rats. Interestingly, GFAP (glial fibrillary associated protein) positive cells were slightly increased (data not shown), while PSA-NCAM (polysialylated neural cell adhesion molecule) positive cells were dramatically increased (Figures S6A, S6B) at P31. Both GFAP and PSA-NCAM are markers for hippocampal neural progenitor cells, with the former being the earlier stage [23], [24]. Significantly, we observed an elevated number of mossy fibers that expressed PSA-NCAM (data not shown). These data clearly indicate that the number of proliferating progenitors and subsequent newly-born neurons is considerably increased in the hippocampal subgranular zone (SGZ) of ARA(+) diet-treated rats. We confirmed that most of these proliferating cells went on to differentiate into neurons at 1 (Figure S7A, S7B, S7C, S7D) and 4 (data not shown) weeks later.

### Administration of arachidonic acid augments neurogenesis and ameliorates PPI defects in *Pax6*(+/−) rats

**Figure 4:**
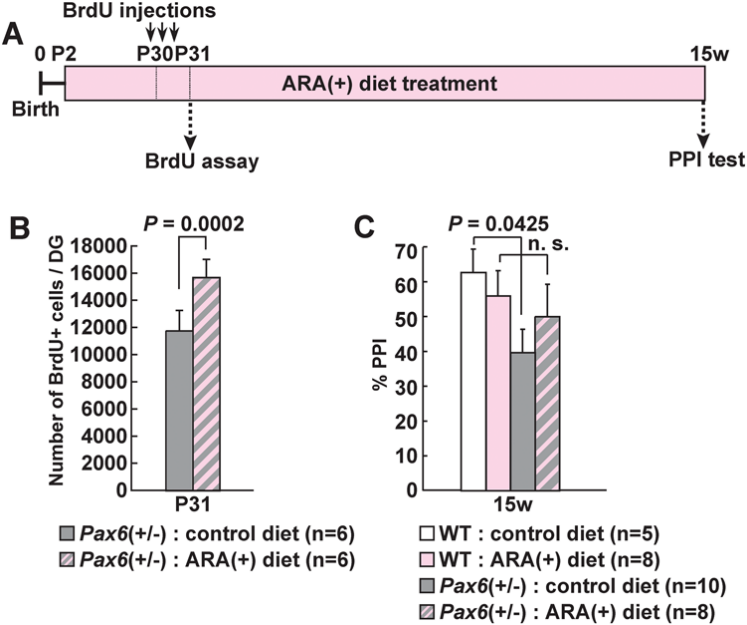
Effects of ARA(+) diets on neurogenesis and PPI in *Pax6*(+/−) rats. (A), Experimental design of the analyses. (B), The number of BrdU-positive cells in the DG of ARA(+) diet-treated *Pax6*(+/−) rats was 33% higher than in control diet-treated *Pax6*(+/−) rats. Student *t*-test was performed and error bars show mean±SE. (C), ARA restored PPI defects in *Pax6*(+/−) rats to a non-significantly different level compared to wild-type rats (the second column vs. the forth column), at pp68 with a startle stimuli of 105 dB. Scheffe's *F* test was performed and error bars show mean±SE. n.s., not significant.

Since dietary ARA improved neurogenesis in normal rats, we embarked on a study to see whether ARA-treatments could restore defective neurogeneis (an approximately 30% decrease) in *Pax6*(+/−) rats [13]. Wild type and *Pax6*(+/−) rat pups were raised on diets either containing or lacking ARA from day P2 to P31 (Figure 4A). When the ARA(+) diet was given to *Pax6*(+/−) rats, neurogenesis was increased by 33% at P31 compared to those fed with control diet (Figure 4B).

These results prompted us to see whether impaired PPI in *Pax6*(+/−) rats could be also reinstated by ARA-treatment, providing a potential therapeutic intervention. The reduced PPI seen in *Pax6*(+/−) rats was partially recovered with an ARA(+) diet at 15 weeks, compared to *Pax6*(+/−) rats fed with a control diet (Figure 4C) [also note that there were no significant differences of PPI between wild-type rats with ARA(+) diet and *Pax6* mutants with ARA(+) diet] (for the results of PPI at pp 71 dB, see Figure S8).

## Discussion

Although PPI has been most frequently studied in schizophrenia, impairments have also been observed in patients with neuropsychiatric disorders including bipolar disorder, autism and Tourette syndrome [25], suggesting that PPI defects may be a shared neural dysfunction across the current diagnostic categories of mental disorders. Therefore, in this study, we targeted PPI as a behavioral phenotype that is highly relevant to psychiatric illnesses and may constitute an important aspect of the pathophysiology of these diseases.

In this study, we have shown that postnatal neurogenesis can be impaired by an environmental milieu, MAM-treatments, in addition to gene disruptions (*Pax6* in the current study) in rats. In addition, we reveal for the first time that even postnatal MAM administration can induce PPI deficits. It is already known that the MAM-induced decrease in fetal neurogenesis can cause PPI deficits [16]. The current findings also suggest that once postnatal neurogenesis is impaired, at the developmental period equivalent to adolescence in humans even if neurogenesis recovers later in life, the susceptibility for PPI deficits remains. This may mean that a ‘critical period’ for the formation of neural circuits underlying PPI spans at least 4–5 weeks in rats. The 4–5 week-old rats show salient neurogenesis [13]. However, because we did not examine other developmental periods for MAM-treatments, we recognize the need for further studies to determine the chronological relationship between impaired neurogenesis and reduced PPI.

It is noteworthy that the administration of ARA successfully and dramatically increased neurogenesis not only in wild-type but also *Pax6*(+/−) rats. It is known that prostaglandin E2 (PGE2) plays an important role in neurogenesis [26], stimulates CB2, a receptor for endocannabinoids and promotes mouse neural stem cell proliferation [27]. Both PGE2 and endocannabinoids are metabolites of ARA. It is also possible that ARA itself augments proliferation of neural progenitor cells. ARA could be transmitted from Fabps to nuclear receptor proteins [17], thereby indirectly controlling the transcription of genes related to cell proliferation. This scenario is analogous to that where retinoic acid (a metabolite of vitamin A) is transmitted from a cellular retinoic acid binding protein and Fabp5 to nuclear receptors RAR and PPARβ/δ, respectively [28]. We also speculate from our current study that the effect of DHA albeit small, may be caused by promoting differentiation and prevention of apoptosis, rather than by increasing cell proliferation [Figures S7B, S7D] [29]. The precise mechanisms for the role of PUFAs in fostering neurogenesis, however, warrants further studies.

Treating the *Pax6*(+/−) rats with ARA for 15 weeks from birth, we were able to alleviate their PPI defects. Because this was the first attempt to evaluate the effects of ARA on PPI, we administered ARA long term to obtain an unequivocal and maximum outcome. There are several possibilities of how ARA can prevent or restore PPI defects in our model rodents. One explanation is that ARA affects postnatal neurogenesis in several brain regions including the hippocampus, and eventually leads to modifications of the neural circuitry of PPI [see Figure S1 and Table S2]. It may not be excluded another explanation, for example, that ARA can directly influence the fluidity of neuronal membranes, thereby regulating neuronal transmission [17], [30] because ARA was continuously administered until scoring PPI. In this case, we may have to consider the roles of Fabp3 also. Fabp3 is expressed in mature neurons in the dentate gyrus (our unpublished results) and reported to have a substantial affinity to ARA [31].

On one hand, the recovery of PPI was not complete in the *Pax6*(+/−) rats. It is likely that the multiple neuro-functional disturbances caused by the gene defect cannot be fully compensated for by postnatal nutritional interventions. In contrast to Mendelian disorders that are usually caused by single gene defects and often manifest with serious physical and/or intellectual abnormalities without effective cures, predisposition to functional psychiatric illnesses is generally associated with multiple susceptibility gene variants with small to modest effects [32]. Therefore, we believe that the improved PPI demonstrated in this study (albeit not complete in gene-disrupted rats), could be meaningful when developing risk-reducing measures for mental disorders.

There are numerous clinical studies reporting symptomatic improvements of schizophrenia and other mental illnesses including mood disorders when PUFAs are given in combination with psychotropic drugs, although PUFAs alone are not sufficient as therapeutic agents [33]–[36]. Such PUFAs are exclusively n-3 fatty acids like EPA (eicosapenteanoic acid) and DHA. The currently observed beneficial effects of ARA may due to the period of administration, i.e. an early postnatal stage, which is different from those examined in human studies. Interestingly it has been reported that patients with schizophrenia show a reduced niacin (vitamin B3) skin flush response, although there is wide inter-individual variation [37]. In this test, varying concentrations of niacin are applied to forearm skin and the flush response is rated [38]. A decreased flush response is deemed to be a peripheral marker of disturbed lipid-ARA pathways [39].

There are two large epidemiological studies demonstrating that when pregnant mothers suffered from famine, the risk of their offspring developing schizophrenia increased to two fold [40], [41]. As a potential mechanism, Heijman et al. recently reported that individuals who were prenatally exposed to famine in Holland had, 6 decades later, less DNA methylation of an imprinted gene compared with their unexposed, same-sex siblings [42]. Therefore, relationship between ARA treatments (and their timing) and epigenetic changes would make a compelling topic for future research. Although multiple nutrients are needed for healthy brain development and maintenance, our current results suggest that an adequate maternal dietary intake of ARA may be especially important for reducing the vulnerability of brains to mental illnesses.

Dietary ARA is a PUFA that can be transmitted directly into the brain via the blood-brain barrier and occurs naturally in nourishing foods such as meat, eggs, fish and seaweed. ARA would therefore appear to be a beneficial molecule for promoting neurogenesis and PPI integrity in a safer way than many artificial compounds. We hope that future studies address the possibility that PUFAs given to lactating mothers (and to young children up to puberty) may reduce the burden of psychiatric diseases including schizophrenia [43], [44]. These studies should clarify the untested issues including the optimal ratio of *n-3* and *n-6*, the total dosage of PUFAs, the time of intervention and period of administration.

## Supporting Information

Figure S1 Schematic model of neural substrates regulating acoustic startle and PPI. The neural circuit is based on reference [43]. In the hippocampus (HPC) neurogenesis occurs throughout life (but prominent in early postnatal period). The nucleus accumbens (NAC) is a pivotal anatomical substrate in the circuit, and there is a report demonstrating that postnatal neurogenesis occurs also in this brain region until P7 [47]. The abbreviations are: Aud, auditory cortex; BLA, basolateral amygdala; Coch, cochlea; IC, inferior colliculus; MPFC, medial prefrontal cortex; MS, medial septal nucleus; NAC, nucleus accumbens; HPC, hippocampus; MD, mediodorsal thalamus; PnC, nucleus reticularis pontis caudalis; PPTg, pedunculopontine nucleus; SC, superior colliculus; VP, ventral pallidum; VTA, ventral tegmental area.
(0.84 MB TIF)

Figure S2 Startle amplitude according to age. The startle amplitude in response to 65, 68, 71, 77, 80, 83, 89, or 95 dB stimuli was measured in both the wild-type and Pax6(+/−) rats. There were no differences between the two groups of rats. Student t-test was performed and error bars show mean±SE. n.s., not significant between the two groups of rats at each startle stimulus.
(1.31 MB TIF)

Figure S3 PPI in MAM-treated wild type rats. (A) Experimental design for MAM treatments and PPI test in wild-type rats. (B, C) PPI defects were observed at pp71 dB with a startle sound of 105 dB, and at pp68 and 71 with startle stimulus of 120 dB at 10 week in the MAM-treated rats. Fisher's PLSD test was performed and error bars show mean±SE.
(1.40 MB TIF)

Figure S4 Expression patterns of Fabp7 and Fabp5 in the DG of wild-type rats. Previous studies have reported that Fabp7 is expressed in the subgranular zone (SGZ) of the hippocampal DG, and is co-expressed with GFAP [48], a marker for astrocytes and neural stem/early progenitor cells. It has also been shown that Fabp5, another member of the fatty acid binding protein family, is expressed in the hippocampus [20]. However, to date no detailed information has been available on cell types that are positive for Fabp7 and Fabp5 during hippocampal neurogenesis. In this study, we revealed for the first time the cell types that express Fabp7 and Fabp5 in the hippocampus by using immunostaining methodology. (A, B) Many Fabp7 positive and Fabp5 positive cells are observed in the subgranular zone (SGZ) and hilus of hippocampus at postnatal day 28 (P28). A much smaller number of Fabp7 and Fabp5 positive cells were detected in the hilus and the molecular layer, with very few cells in the granule cell layer (GCL). Fabp7 and Fabp5 positive cells were highly proliferative; all cells incorporated BrdU within 1 day (data not shown). The pattern and morphology of Fabp7 positive cells differed slightly from Fabp5 positive cells, in that Fabp7 positive cells had long, thin projections and were present as individual cells (A inset), while Fabp5 positive cells formed clusters (B inset). These findings suggest that Fabp7 positive cells are slightly more primitive neural progenitor cells compared to Fabp5 positive cells. (C–E) We performed double staining analyses using various markers for neural stem/progenitor cells, neurons and astrocytes. (C, E) Many Fabp7 positive cells (65.0%) co-expressed an early progenitor marker GFAP, and the majority of Fabp7/GFAP double positive cells exhibited processes that were oriented radially into the GCL of the DG. Fabp7 positive cells also expressed a neural stem cell marker nestin. About a third of Fabp7 positive cells (40.4%) co-expressed a late progenitor marker, PSA-NCAM. However, Fabp7 positive cells rarely expressed the granule cell marker calbindin D-28k (4.8%). In addition, the ratio of Fabp7 positive cells to the total of BrdU positive cells was 100% in the DG of hippocampus. (D, E), A smaller proportion of Fabp5 positive cells (38.8%) co-expressed GFAP, and the majority of Fabp5 positive cells were distributed as clusters, in contrast to Fabp7 positive cells (A, B). Moreover, many of the Fabp5 positive cells co-expressed PSA-NCAM (59.7%), and some of Fabp5 positive cells co-expressed nestin. A slightly higher proportion of Fabp5 positive cells expressed calbindin D-28k (15.4%) than Fabp7 positive cells. Fabp7 and Fabp5 were often co-expressed in the same cells (data not shown). (F) Schematic illustration of the expression patterns of Fabp7 and Fabp5. Fabp7 and Fabp5 positive cells have neural stem/progenitor cell like properties, and that the former showed slightly more primitive characteristics than the latter.
(9.61 MB TIF)

Figure S5 Transition of PUFAs into the brains of rat pups. The ratio of ARA/DHA in the hippocampus of ARA(+) diet-treated rat pups is higher, and conversely lower in DHA(+) diet-treated pups, compared to that of control diet-treated rats. Because pups are totally dependent on maternal breast milk of their mothers would have ingested PUFAs and secreted them into their milk, pups can take ARA or DHA from breast milk at the early postnatal stage. After 3 weeks, pups also eat the food containing PUFAs by themselves. Therefore, PUFAs are thought to be continuously transferred to the brain of pups until P31. Scheffe's F test was performed and error bars show mean±SE.
(0.66 MB TIF)

Figure S6 Identification of neural progenitor cells in the DG of ARA(+) diet-treated wild-type rats. (A) Experimental design for ARA(+) diet-diet-treatment. Rat pups and their mothers are fed with two different diets [control or ARA(+)] from P2 to P31. They were sacrificed at P31. (B) The number of PSA-NCAM positive cells was increased in the DG of the ARA(+) diet-treated wild-type rats compared to control diet-treated rats.
(4.55 MB TIF)

Figure S7 Cell fate analysis of newborn cells in the DG of PUFA-treated rats. (A) Experimental design for survival and cell fate assays in the PUFA- treated rats. The PUFA-treated rats were injected with BrdU three times at P30 and sacrificed at P31 or P37. (B) Calculated survival rate and differentiation rate of BrdU-positive cells in the DG 7 days after BrdU-injection at P30. The survival rate was not differed between control diet-treated wild-type rats (131%) and ARA(+) diet-treated wild-type rats (132%) (see the fourth column), while it was reduced in DHA(+) diet-treated wild-type rats (114%) and to lesser extent in ARA(+)DHA(+) diet-treated rats (123%), compared with control diet treated wild-type rats. The percentage of NeuN positive cells in total BrdU positive cells corresponds to the degree of production of newborn neurons. The frequency of NeuN positivity in total BrdU+ cells was lower in ARA(+) diet-treated wild-type rats (51%) but higher in DHA(+) diet-treated wild-type rats (66%) compared with control diet treated wild-type rats (see the fifth column). (C) A BrdU labeled cell expressing NeuN at 7 days after BrdU injection in a control diet-treated wild-type rat. (D) Estimated numbers of newborn neurons that were double positive for BrdU and NeuN, were calculated using the survival rate and the differentiation rate ( = the third column value×the fifth column value in B). The estimated total number of newly generated neurons of ARA(+) diet-treated rats examined at P37 showed a trend of increase, although statistically not significant, compared to those from the control diet-treated rats. Although the DHA(+) diet-treated rats showed a comparable increase of newly generated neurons to that of the ARA(+) diet-treated rats, this increase is thought to be due to the accelerated differentiation to NeuN positive cells. That is, a relatively high ratio of (NeuN+)&(BrdU+)/total BrdU+ cells (the fifth column in B) indicates that DHA promotes neuronal differentiation rather than production of progenitor cells. In addition, the relatively low ratio of P37/P31 BrdU+ cells (114%) (the fourth column in B) in the DHA(+) diet-treated rats is consistent with the above theory, because later lineages of progenitor cells have a more limited number of cell divisions. The number of newly generated neurons in ARA(+)DHA(+) diet-treated rats was not changed compared with that of control diet-treated rats. This might suggest that ARA and DHA impinge on the generation of new neurons in an antagonistic manner.
(1.06 MB TIF)

Figure S8 Effects of ARA(+) diets on PPI in Pax6(+/−) rats. (A), Experimental design of the analyses. (B), PPI was scored at pp71. There were no significant differences of PPI among any groups, but similar trends were observed as those in Figure 4C. Scheffe's F test was performed and error bars show mean±SE.
(0.87 MB TIF)

Text S1 Materials and Methods for supporting information
(0.02 MB DOC)

Table S1 List of primary and secondary antibodies used in the current study.
(0.02 MB XLS)

Table S2 Summary of relationship between postnatal neurogenesis and PPI shown in this study.
(0.01 MB XLS)
