# Supplementary figures and images for: Arachidonic Acid Drives Postnatal Neurogenesis and Elicits a Beneficial Effect on Prepulse Inhibition, a Biological Trait of Psychiatric Illnesses
Source: PLoS One. 2009 Apr 8;4(4):e5085. doi: 10.1371/journal.pone.0005085 (PMC2663848; doi:10.1371/journal.pone.0005085)

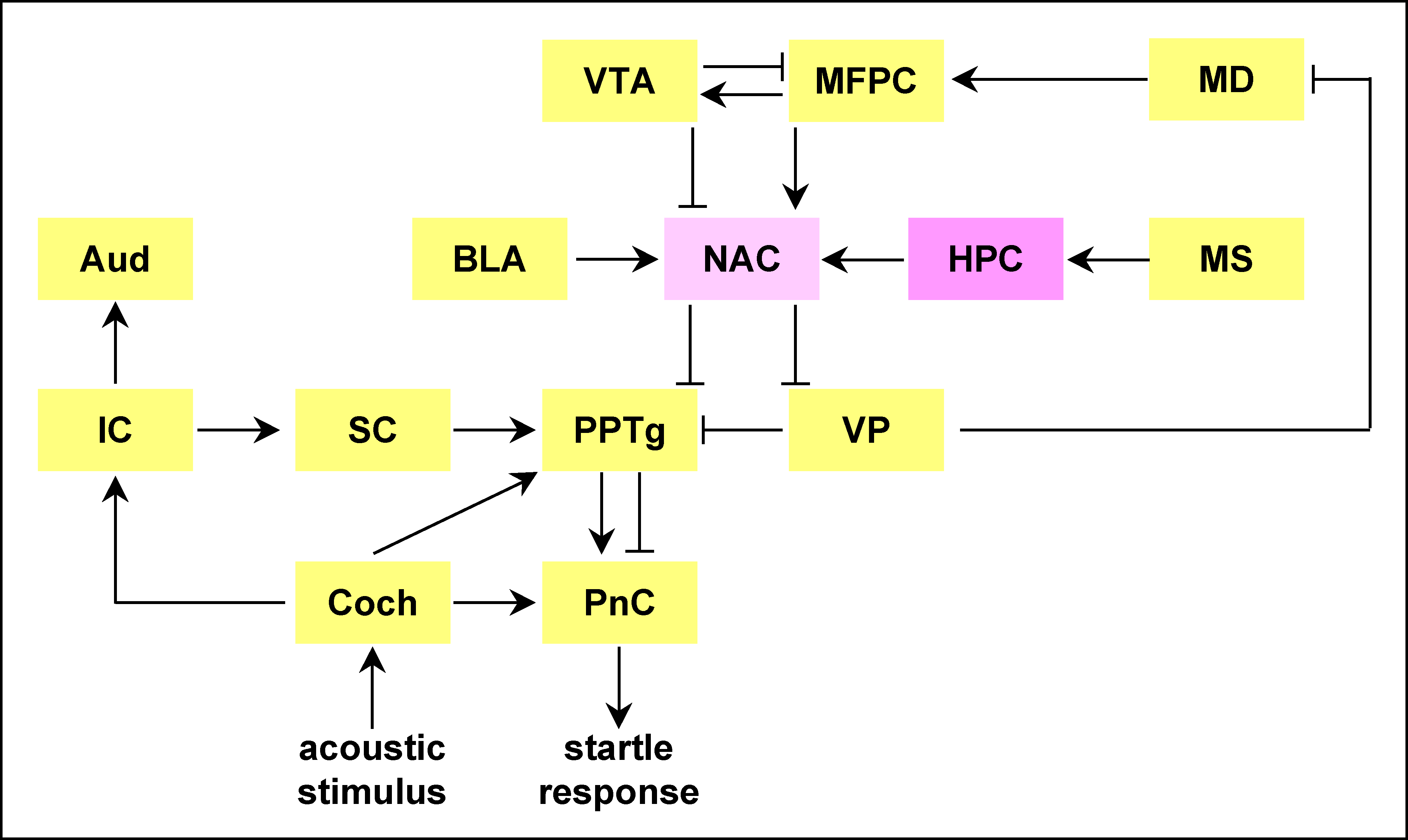

Supplement: Figure S1 — Schematic model of neural substrates regulating acoustic startle and PPI. The neural circuit is based on reference [43]. In the hippocampus (HPC) neurogenesis occurs throughout life (but prominent in early postnatal period). The nucleus accumbens (NAC) is a pivotal anatomical substrate in the circuit, and there is a report demonstrating that postnatal neurogenesis occurs also in this brain region until P7 [47]. The abbreviations are: Aud, auditory cortex; BLA, basolateral amygdala; Coch, cochlea; IC, inferior colliculus; MPFC, medial prefrontal cortex; MS, medial septal nucleus; NAC, nucleus accumbens; HPC, hippocampus; MD, mediodorsal thalamus; PnC, nucleus reticularis pontis caudalis; PPTg, pedunculopontine nucleus; SC, superior colliculus; VP, ventral pallidum; VTA, ventral tegmental area. (0.84 MB TIF) [file pone.0005085.s001.tif]

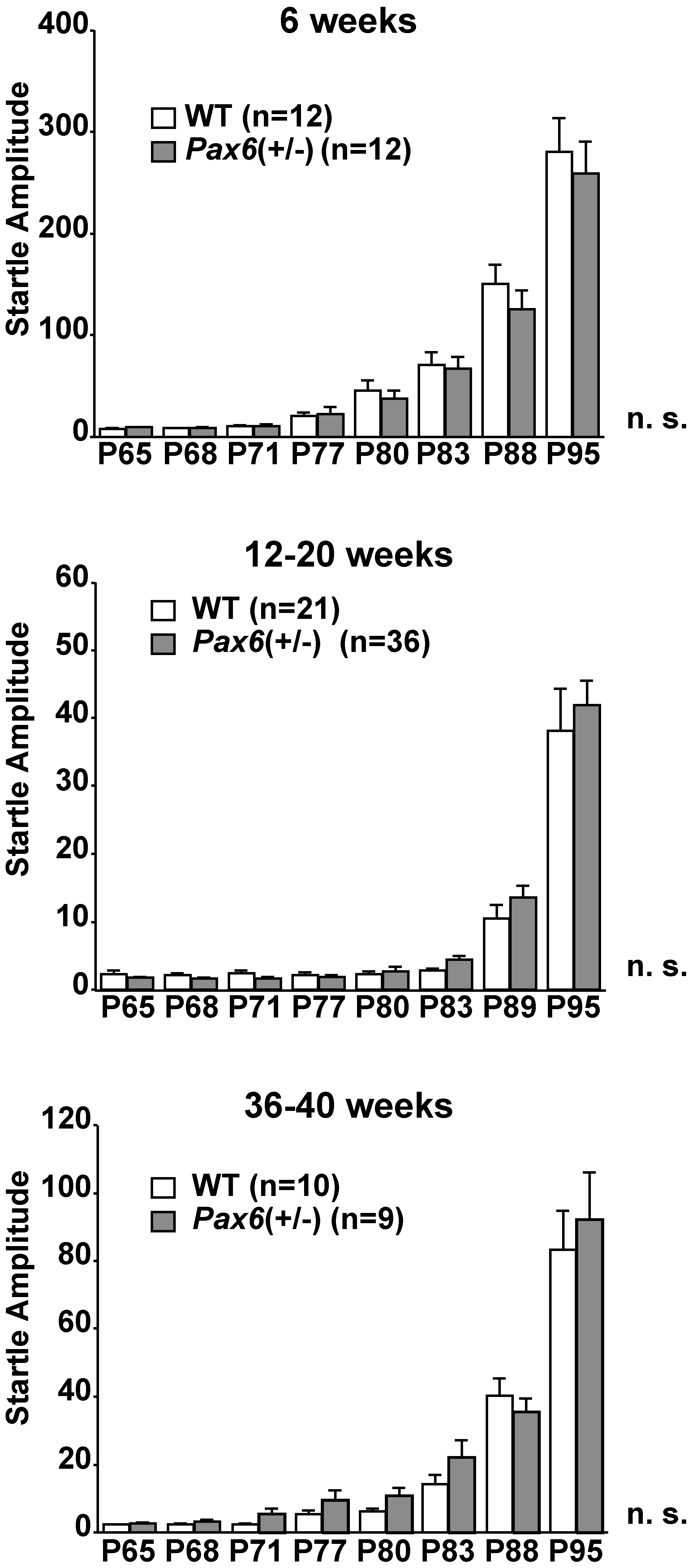

Supplement: Figure S2 — Startle amplitude according to age. The startle amplitude in response to 65, 68, 71, 77, 80, 83, 89, or 95 dB stimuli was measured in both the wild-type and Pax6(+/−) rats. There were no differences between the two groups of rats. Student t-test was performed and error bars show mean±SE. n.s., not significant between the two groups of rats at each startle stimulus. (1.31 MB TIF) [file pone.0005085.s002.tif]

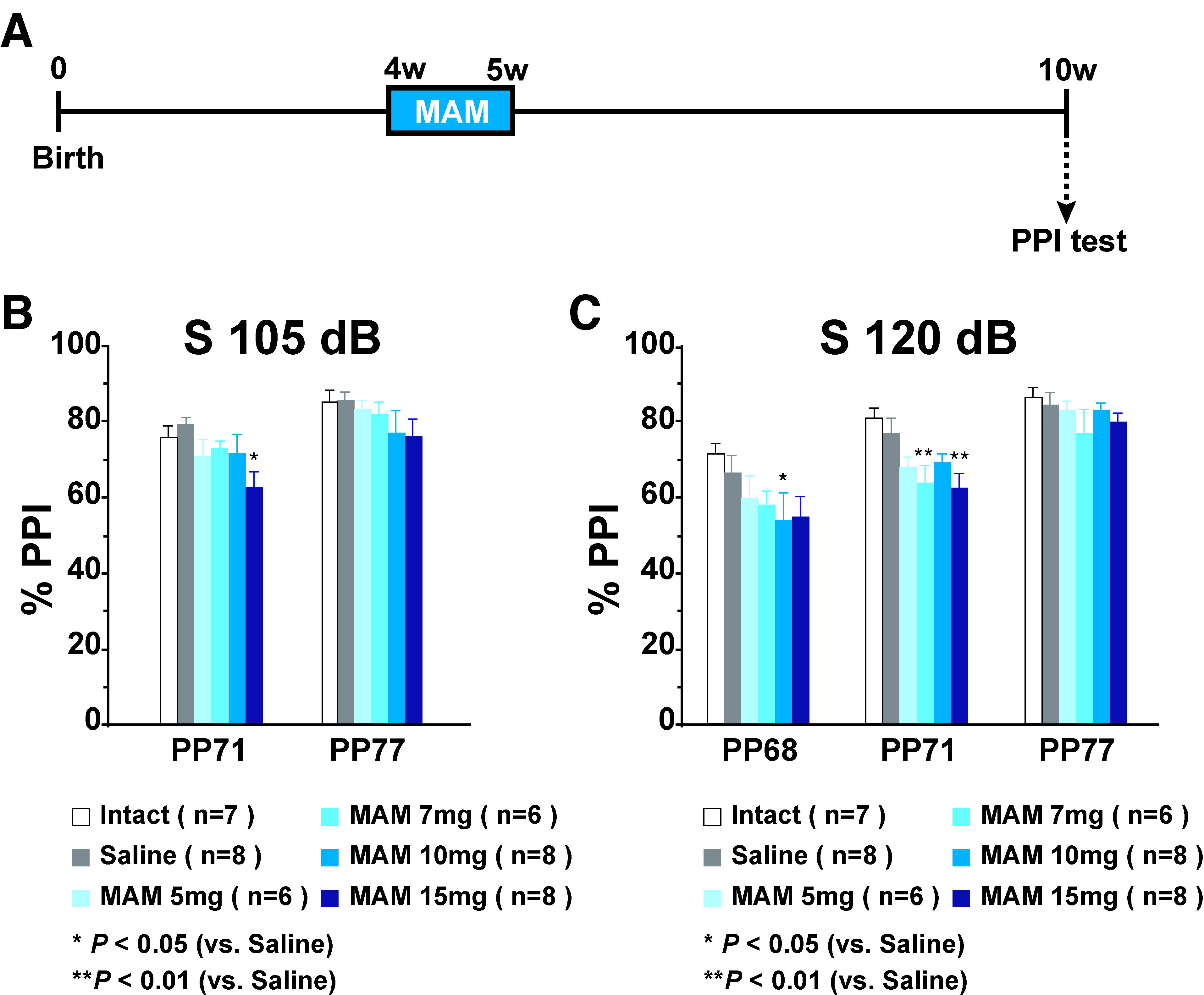

Supplement: Figure S3 — PPI in MAM-treated wild type rats. (A) Experimental design for MAM treatments and PPI test in wild-type rats. (B, C) PPI defects were observed at pp71 dB with a startle sound of 105 dB, and at pp68 and 71 with startle stimulus of 120 dB at 10 week in the MAM-treated rats. Fisher's PLSD test was performed and error bars show mean±SE. (1.40 MB TIF) [file pone.0005085.s003.tif]

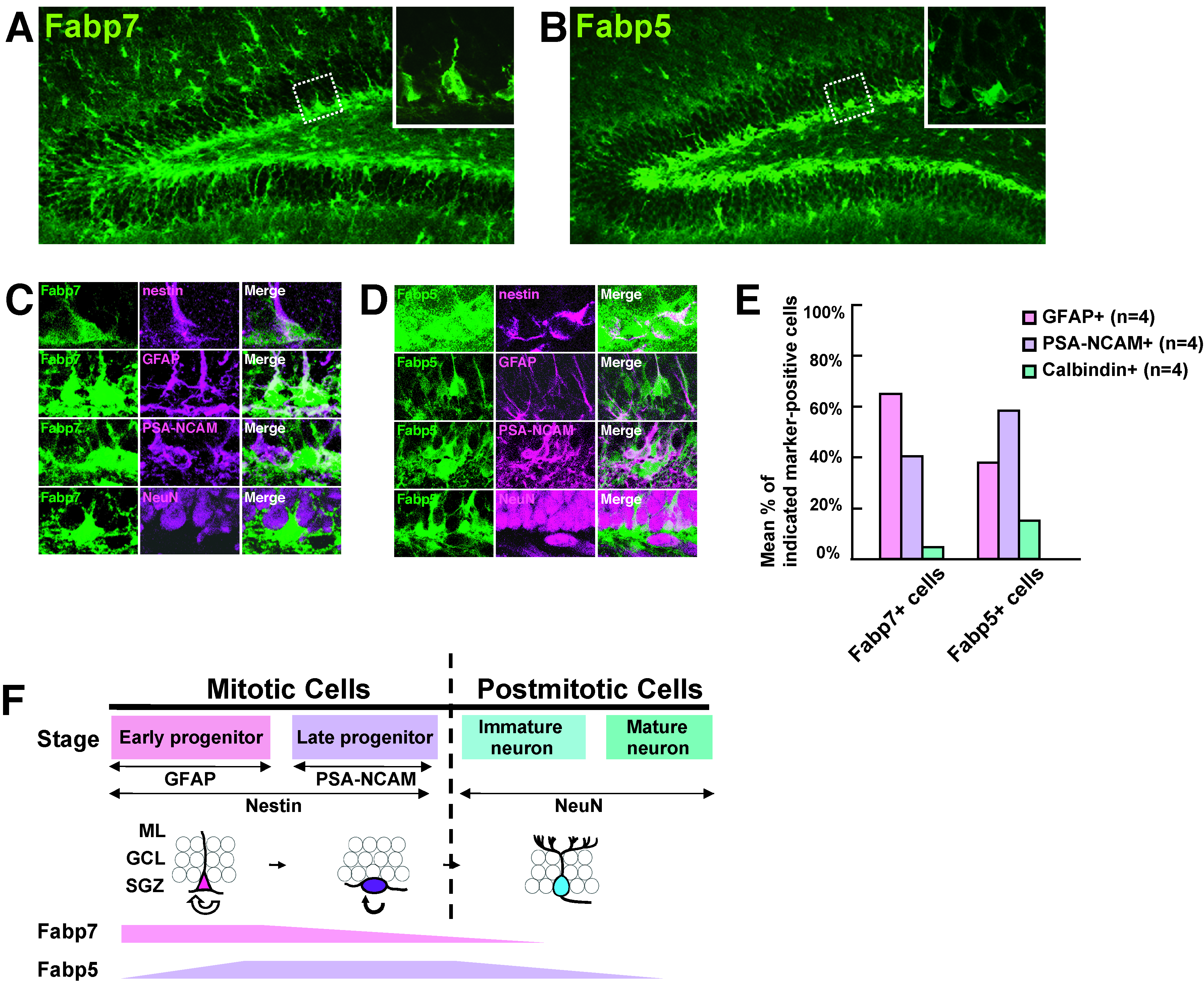

Supplement: Figure S4 — Expression patterns of Fabp7 and Fabp5 in the DG of wild-type rats. Previous studies have reported that Fabp7 is expressed in the subgranular zone (SGZ) of the hippocampal DG, and is co-expressed with GFAP [48], a marker for astrocytes and neural stem/early progenitor cells. It has also been shown that Fabp5, another member of the fatty acid binding protein family, is expressed in the hippocampus [20]. However, to date no detailed information has been available on cell types that are positive for Fabp7 and Fabp5 during hippocampal neurogenesis. In this study, we revealed for the first time the cell types that express Fabp7 and Fabp5 in the hippocampus by using immunostaining methodology. (A, B) Many Fabp7 positive and Fabp5 positive cells are observed in the subgranular zone (SGZ) and hilus of hippocampus at postnatal day 28 (P28). A much smaller number of Fabp7 and Fabp5 positive cells were detected in the hilus and the molecular layer, with very few cells in the granule cell layer (GCL). Fabp7 and Fabp5 positive cells were highly proliferative; all cells incorporated BrdU within 1 day (data not shown). The pattern and morphology of Fabp7 positive cells differed slightly from Fabp5 positive cells, in that Fabp7 positive cells had long, thin projections and were present as individual cells (A inset), while Fabp5 positive cells formed clusters (B inset). These findings suggest that Fabp7 positive cells are slightly more primitive neural progenitor cells compared to Fabp5 positive cells. (C–E) We performed double staining analyses using various markers for neural stem/progenitor cells, neurons and astrocytes. (C, E) Many Fabp7 positive cells (65.0%) co-expressed an early progenitor marker GFAP, and the majority of Fabp7/GFAP double positive cells exhibited processes that were oriented radially into the GCL of the DG. Fabp7 positive cells also expressed a neural stem cell marker nestin. About a third of Fabp7 positive cells (40.4%) co-expressed a late progenitor marker [file pone.0005085.s004.tif]

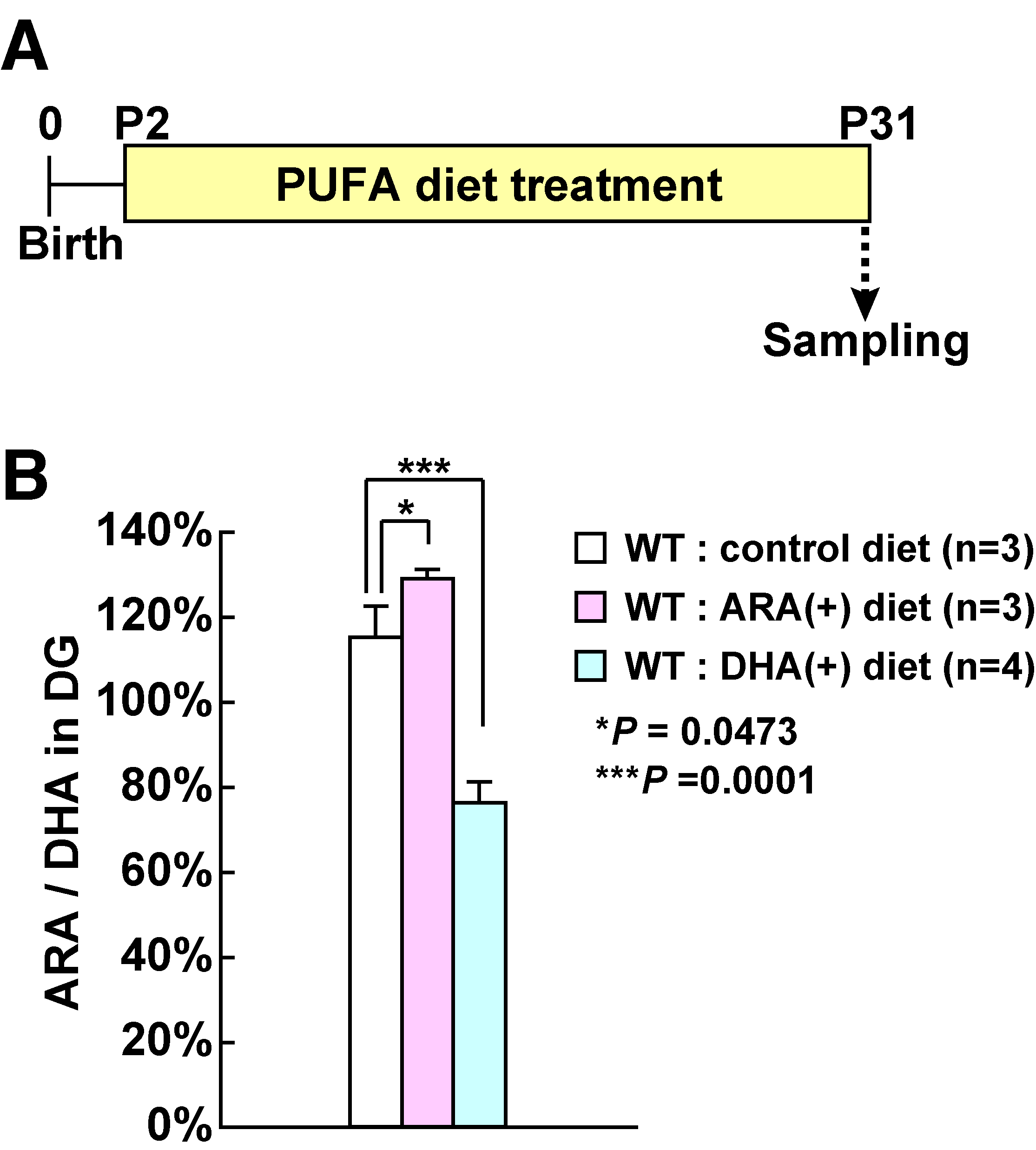

Supplement: Figure S5 — Transition of PUFAs into the brains of rat pups. The ratio of ARA/DHA in the hippocampus of ARA(+) diet-treated rat pups is higher, and conversely lower in DHA(+) diet-treated pups, compared to that of control diet-treated rats. Because pups are totally dependent on maternal breast milk of their mothers would have ingested PUFAs and secreted them into their milk, pups can take ARA or DHA from breast milk at the early postnatal stage. After 3 weeks, pups also eat the food containing PUFAs by themselves. Therefore, PUFAs are thought to be continuously transferred to the brain of pups until P31. Scheffe's F test was performed and error bars show mean±SE. (0.66 MB TIF) [file pone.0005085.s005.tif]

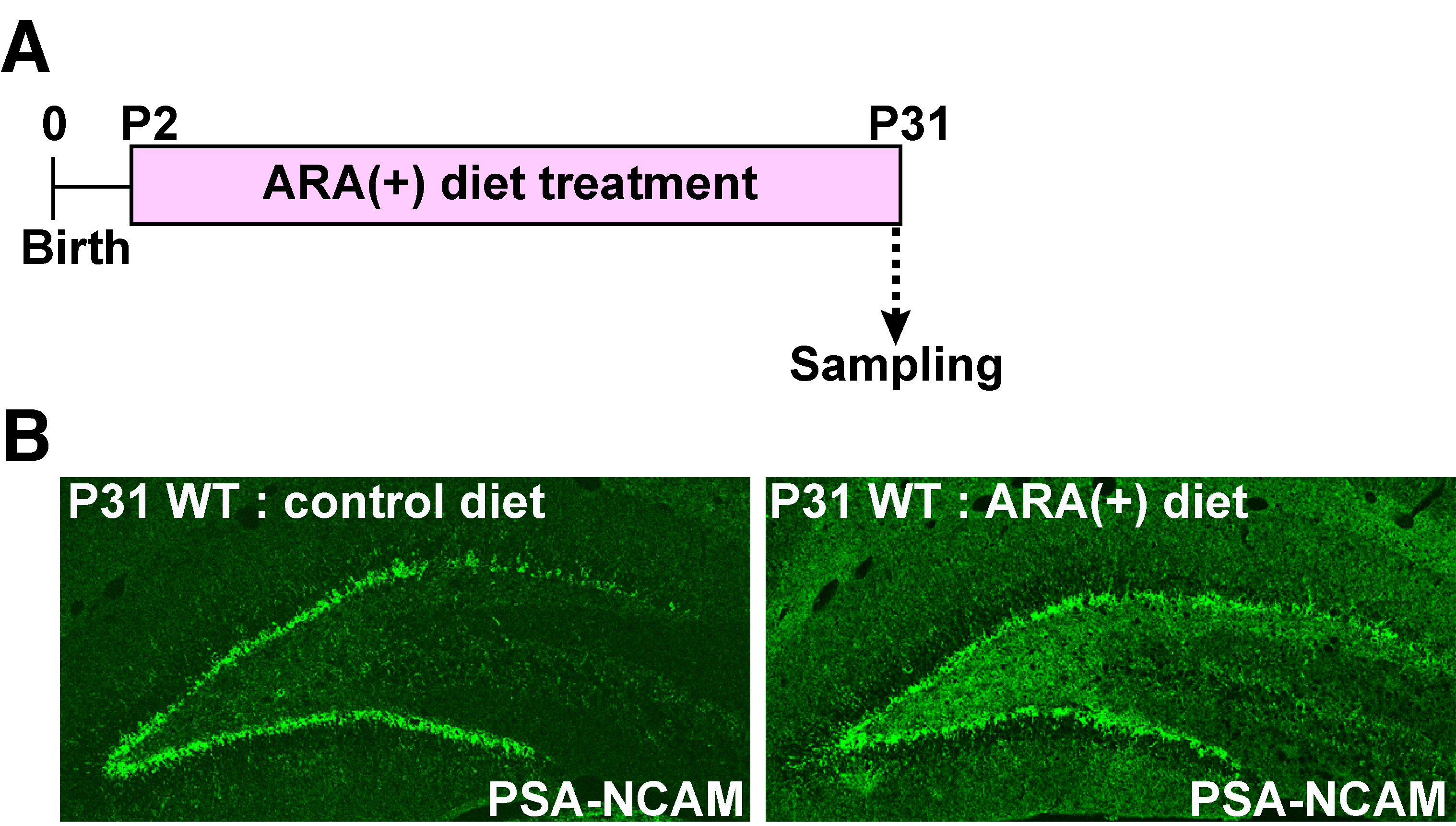

Supplement: Figure S6 — Identification of neural progenitor cells in the DG of ARA(+) diet-treated wild-type rats. (A) Experimental design for ARA(+) diet-diet-treatment. Rat pups and their mothers are fed with two different diets [control or ARA(+)] from P2 to P31. They were sacrificed at P31. (B) The number of PSA-NCAM positive cells was increased in the DG of the ARA(+) diet-treated wild-type rats compared to control diet-treated rats. (4.55 MB TIF) [file pone.0005085.s006.tif]

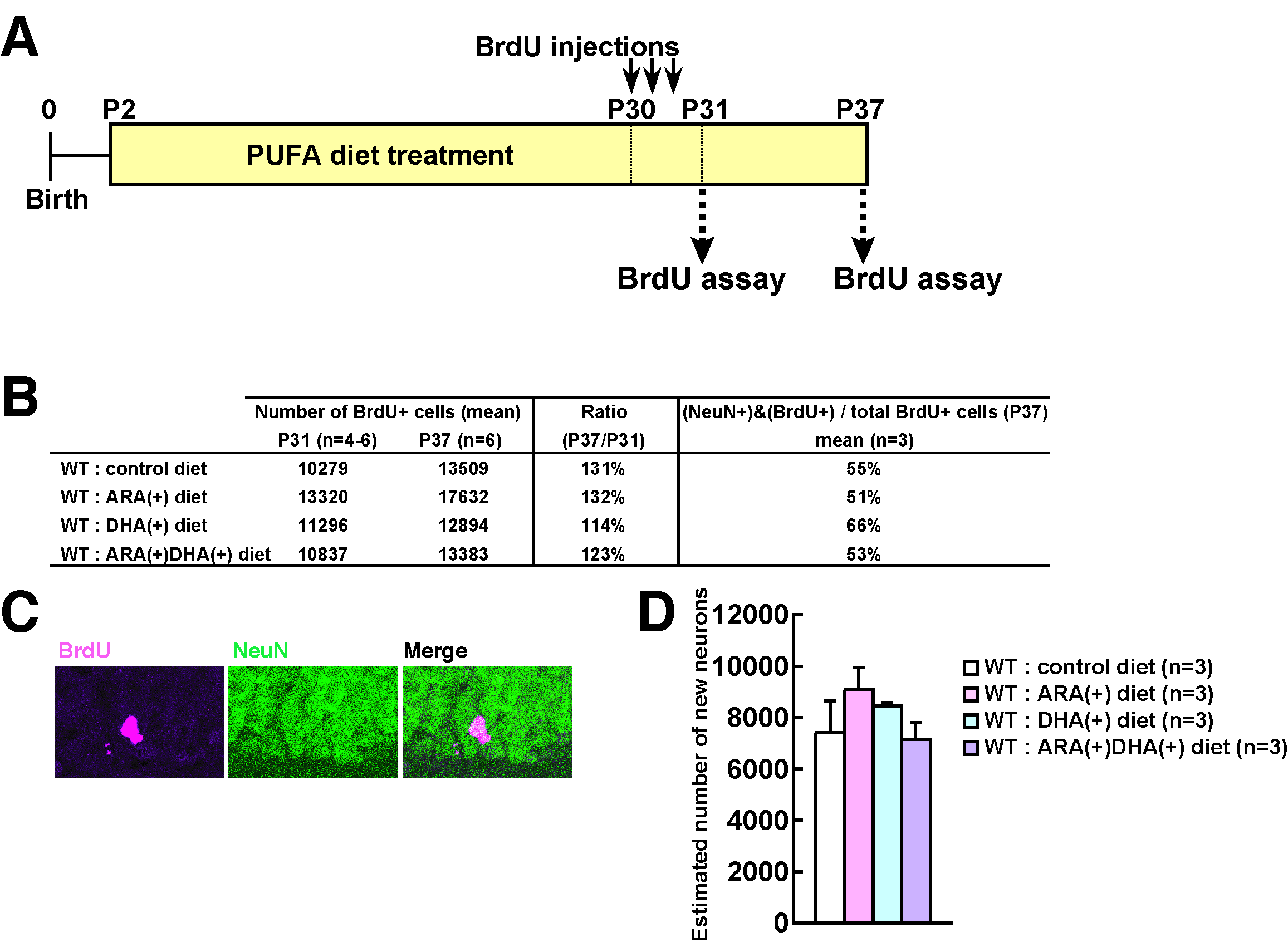

Supplement: Figure S7 — Cell fate analysis of newborn cells in the DG of PUFA-treated rats. (A) Experimental design for survival and cell fate assays in the PUFA- treated rats. The PUFA-treated rats were injected with BrdU three times at P30 and sacrificed at P31 or P37. (B) Calculated survival rate and differentiation rate of BrdU-positive cells in the DG 7 days after BrdU-injection at P30. The survival rate was not differed between control diet-treated wild-type rats (131%) and ARA(+) diet-treated wild-type rats (132%) (see the fourth column), while it was reduced in DHA(+) diet-treated wild-type rats (114%) and to lesser extent in ARA(+)DHA(+) diet-treated rats (123%), compared with control diet treated wild-type rats. The percentage of NeuN positive cells in total BrdU positive cells corresponds to the degree of production of newborn neurons. The frequency of NeuN positivity in total BrdU+ cells was lower in ARA(+) diet-treated wild-type rats (51%) but higher in DHA(+) diet-treated wild-type rats (66%) compared with control diet treated wild-type rats (see the fifth column). (C) A BrdU labeled cell expressing NeuN at 7 days after BrdU injection in a control diet-treated wild-type rat. (D) Estimated numbers of newborn neurons that were double positive for BrdU and NeuN, were calculated using the survival rate and the differentiation rate ( = the third column value×the fifth column value in B). The estimated total number of newly generated neurons of ARA(+) diet-treated rats examined at P37 showed a trend of increase, although statistically not significant, compared to those from the control diet-treated rats. Although the DHA(+) diet-treated rats showed a comparable increase of newly generated neurons to that of the ARA(+) diet-treated rats, this increase is thought to be due to the accelerated differentiation to NeuN positive cells. That is, a relatively high ratio of (NeuN+)&(BrdU+)/total BrdU+ cells (the fifth column in B) indicates that DHA promotes neuronal differentiation rather t [file pone.0005085.s007.tif]

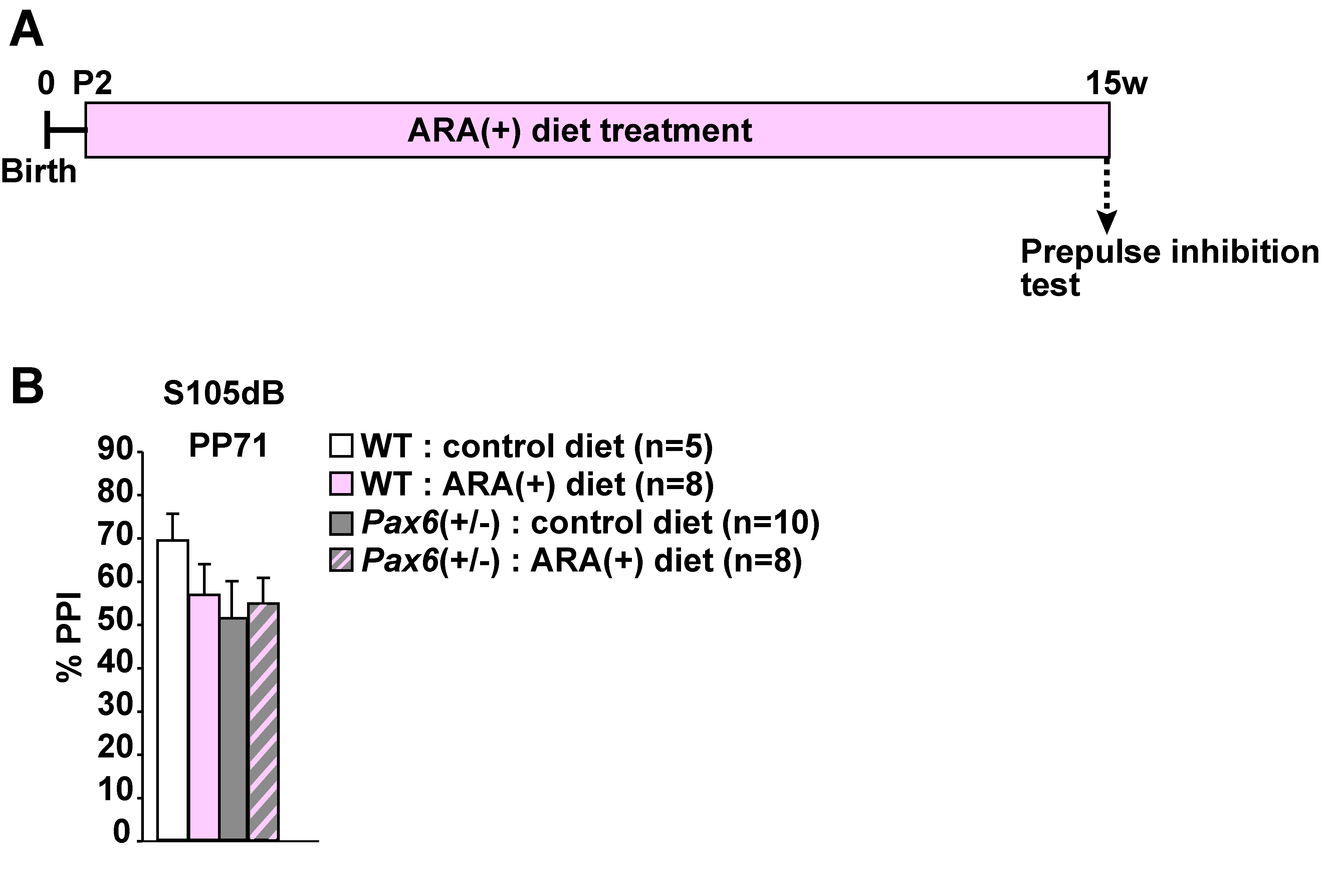

Supplement: Figure S8 — Effects of ARA(+) diets on PPI in Pax6(+/−) rats. (A), Experimental design of the analyses. (B), PPI was scored at pp71. There were no significant differences of PPI among any groups, but similar trends were observed as those in Figure 4C. Scheffe's F test was performed and error bars show mean±SE. (0.87 MB TIF) [file pone.0005085.s008.tif]
