# Supplementary material for: Arachidonic Acid Drives Postnatal Neurogenesis and Elicits a Beneficial Effect on Prepulse Inhibition, a Biological Trait of Psychiatric Illnesses
Source: PLoS One. 2009 Apr 8;4(4):e5085. doi: 10.1371/journal.pone.0005085 (PMC2663848; doi:10.1371/journal.pone.0005085)
Supplement: Text S1 — Materials and Methods for supporting information (0.02 MB DOC) [file pone.0005085.s009.doc]

# Text S1: Materials and Methods for supporting information

**Fatty acid analysis**

Total lipids in the hippocampal dentate gyrus were extracted using chloroform and methanol [45]. The phospholipids fraction was separated from the extracted lipids by thin-layer chromatography using silica gel 60 (Merck, Darmstadt, Germany). The solvent system used was n-hexane/diethyl ether (70/30, v/v). The fatty acid residues in the phospholipids fraction were analyzed [46]. In brief, an internal standard (pentadecanoic acid) was added to each sample and it was incubated in methanolic HCl at 50°C for 3 hr to induce transmethylation of fatty acid residues. The fatty acid methyl esters were extracted with n-hexane and analyzed using capillary gas-liquid chromatography. Analytical conditions were as follows: 1) apparatus: Agilent 6890 (Agilent, Delaware); 2) column, SP-2330 (30 m x 0.32 mm x 0.2 mm, SUPELCO, Pennsylvania); 3) carrier: He (30 cm/s); 4) column temperature: 180°C (2 min) followed by +2°C /min to 220°C.
